# Morphological and molecular development of *Terfezia claveryi* ectendomycorrhizae exhibits three well-defined stages

**DOI:** 10.1007/s00572-025-01205-8

**Published:** 2025-04-15

**Authors:** Ángel Luigi Guarnizo, José Eduardo Marqués-Gálvez, Francisco Arenas, Alfonso Navarro-Ródenas, Asunción Morte

**Affiliations:** Departamento Biología Vegetal, Facultad de Biología, CEIR Campus Mare Nostrum (CMN), Universidad de Murcia, Campus de Espinardo, 30100 Murcia, Spain

**Keywords:** Mycorrhizal symbiosis, Aquaporin, Desert truffles, Pre-symbiosis

## Abstract

The normal development of mycorrhizal symbiosis is a dynamic process, requiring elaborately regulated interactions between plant roots and compatible fungi, mandatory for both partners´ survival. In the present study, we further elucidated the mycorrhizal development of the desert truffles *Terfezia claveryi* with the host plant *Helianthemum almeriense* as an ectendomycorrhizal symbiosis model under greenhouse conditions. To investigate this, we evaluated the morphology of mycorrhizal colonization, concomitantly with the dynamic expression of selected marker genes (6 fungal and 11 plant genes) measured every week until mycorrhiza maturation (three months). We were able to determine 3 main stages in the mycorrhization process, 1) pre-symbiosis stage where mycelium is growing in the soil with no direct interaction with roots, 2) early symbiosis stage when the fungus spreads along the roots intercellularly and plant-fungal signaling is proceeding, and 3) late symbiosis stage where the fungus consolidates and matures with intracellular hyphal colonization; this is characterized by the regulation of cell-wall remodeling processes.

**Supplementary Information:**

The online version contains supplementary material available at 10.1007/s00572-025-01205-8.

## Introduction

Desert truffles are edible hypogeous fruit bodies produced by certain Ascomycete fungi inhabiting arid and semiarid areas. They establish mycorrhizal symbiosis with roots of annual and perennial shrubs belonging to the Cistaceae family (Gutiérrez et al. 2003; Kovács and Trappe 2014; Roth-Bejerano et al. 2014). They are one of the few edible mycorrhizal fungi that have been domesticated (Guerin-Laguette 2021), transforming them in an important resource to enhance the use of arid lands and fight the adverse conditions related to climate change and desertification in the Mediterranean basin and the Middle East (Ferreira et al. 2023). Among them, *Terfezia* spp. are among the most prized species to be cultivated, due to their high nutritional value, delicious taste, anticancer and immunomodulatory activity (Bokhary and Parvez 1993; Al Obaydi et al. 2020; Morte et al. 2021; Veeraraghavan et al. 2021). Desert truffle cultivation faces different agroclimatic challenges in the field (Andrino et al. 2019), but the success of human-managed desert truffle fields first requires nursery plantlet production with enough mycorrhizal development (Morte et al. 2010). The whole process of producing mycorrhizal plantlets is made in three stages (Navarro-Ródenas et al. 2016) and may last from four to eight months and a half, depending on the techniques used (Morte et al. 2017). Mature mycorrhiza is normally observed after eight to twelve weeks, when it reaches a steady state (Morte et al. 2017). *Helianthemum almeriense* x *Terfezia claveryi* mycorrhizal plants are the most used mycorrhizal system for desert truffle cultivation in the Mediterranean area (Morte et al. 2021). They establish ectendomycorrhizal (EEM) symbiosis, which is characterized by the presence of both intercellular hyphae establishing a Hartig Net (HN), similar to the structures observed in ectomycorrhizal fungi (ECM), and intracellular hyphae penetrating the cortex cells and forming a symbiotic surface between the fungal cell wall and the intact host cell plasmalemma (Gutiérrez et al. 2003). They can also present a thin and disorganized fungal mantle surrounding the colonized roots (Dexheimer et al. 1985; Morte et al. 1994; Yu et al. 2001; Gutiérrez et al. 2003; Roth-Bejerano et al. 2014; Louro et al. 2021). Although different morphologies of mycorrhizal root tips have been described for *T. claveryi* (Gutiérrez et al. 2003), its colonization is not limited to root tips but can be found throughout the whole system of fine roots of the host (Navarro-Ródenas et al. 2012a, 2013). In this sense, the EEM symbiosis of *T. claveryi* has previously been described as a *continuum*, a dynamic structure that changes depending on mineral nutrition, water availability or hormonal signal (Gutiérrez et al. 2003; Zaretsky et al. 2006b; Navarro-Ródenas et al. 2012a, 2013; Roth-Bejerano et al. 2014). No morphological time lapse of the initial steps of *H. almeriense* x *T. claveryi* EEM formation has been explored yet and how the mentioned EEM *continuum* switches during the mycorrhizal development in nursery conditions is unknown.

In other mycorrhiza systems, such as ECM, the normal mycorrhiza development both before (pre-symbiotic) and after (symbiotic) physical contact is associated with an intense molecular crosstalk to determine the outcome of the symbiosis (Marqués-Gálvez et al. 2022). Main actors of this interkingdom crosstalk include fungal effectors (Plett et al. 2011, 2020; Kloppholz et al. 2011; Kang et al. 2020), both fungal and plant carbohydrate active enzymes (CAZymes) in charge of the plant cell wall remodeling (Veneault-Fourrey et al. 2014) and other genes related to response to biotic stimulus and signaling and hormone regulation (Labbé et al. 2019; Basso et al. 2020). Transporters are known to play a role in mycorrhiza functioning, as it is the case of the mycorrhizal inducible phosphate transporter PT4 (Harrison et al. 2002) However, other transporters, such as aquaporins (AQP), could also be involved in the first steps of development of the mycorrhiza. For instance, *Laccaria bicolor* AQP1 plays a key role for the establishment of ECM symbiosis, since knocked-down mutants are impaired in HN formation (Navarro-Ródenas et al. 2015). Just as the morphological development of *T. claveryi* EEM is poorly understood, so too are the molecular mechanisms associated to this process. During the preinfection stage between *Terfezia boudieri* and *Helianthemum sessiliflorum*, high concentrations of indole- 3-acetic acid (IAA) secreted by the fungus induced lateral root formation (Sitrit et al. 2014; Turgeman et al. 2016). In the *T. boudieri* x *Cistus incanus* symbiosis, certain genes associated with several signal transduction pathways, could be linked to the regulation of hyphal proliferation and adaptive modifications (Zaretsky et al. 2006a). After contact, during root colonization between *T. claveryi* and *H. almeriense*, a correlation between the fungal aquaporin *TcAQP1* expression and the degree of mycorrhization has been observed (Navarro-Ródenas et al. 2012b). A recent fungal and plant transcriptome analysis of *H. almeriense* x *T. claveryi* has enabled new information about putative mechanisms implicated in the development of the desert truffle EEM symbiosis (Marqués-Gálvez et al. 2021). Most of the molecular markers of mycorrhizal molecular crosstalk mentioned above, including fungal putative effectors (also known as mycorrhiza-induced small secreted proteins or MiSSPs), plant and fungal CAZymes, the fungal aquaporin TcAQP1, and other genes related to pathogenesis response, signaling and regulation of hormonal pathways, were documented, suggesting commonalities with better studied ECM systems.

In the present study, we aim to further describe the morphological development of desert truffle EEM. We hypothesized that, as with other mycorrhizal interactions, *H. almeriense* x *T. claveryi* EEM presents well differentiated morphological stages during its development. To explore this, *H. almeriense* plants were inoculated with *T. claveryi* in greenhouse settings. We evaluated the morphology of mycorrhizal colonization together with the expression levels of selected genes, including plant and fungal aquaporins and genes previously identified to be related with the mycorrhiza development and highly differentially expressed in previous RNA-seq analysis (Marqués-Gálvez et al. 2021). To provide a temporal scale to the dynamic EEM developmental process, we followed these morphological and molecular traits every week until mycorrhiza maturation and discussed their biological implications.

## Materials and methods

### Plant growth conditions

*H. almeriense* seeds were collected from Zarzadilla de Totana, Lorca, Murcia, Spain (37º 52´ 15.5´´ N 1º 42´ 10.5´´ W), then were scarified, sterilized and sown according to Morte et al. (2008). Two months after germination, seedlings were transferred to larger 300-ml pots and inoculated with *T. claveryi* mature truffle spores. The substrate consisted in a mixture of black peat, vermiculite, and sterilized clay soil (1:1:0.5). Subsequently, 210 mycorrhizal plants were grown in a greenhouse located at the Service of Plant Biotechnology (ACTI) in the University of Murcia. All the plants were irrigated four times a week (1.8 L per plant) with an automated sprinkle irrigation system to maintain plants under well-irrigated conditions until the end of the experimental period. Plant harvest, consisting of six random plants, was initiated one week after inoculation, and was repeated every week, for 10 weeks (for a total of 60 harvested mycorrhizal seedlings).

### Measurement of growth parameter

Each week during harvest, total plant length and relative root surface area were measured using image analysis in ImageJ (Schneider et al. 2012). Plant length was obtained from scaled photographs, while root surface area was estimated from images of cleaned roots processed in binary format using the “Analyze Particles” tool. As measurements were based on 2D images, root surface area is considered a relative value.

### Mycorrhizal characterization

Mycorrhizal types were determined using an Olympus BH2 microscope in six plants each week. The whole root system was thoroughly washed with distilled water and fine roots were collected and split into two fractions. One of the fractions was stained with blue ink following the protocol described by Gutiérrez et al. (2003) and observed *in toto* for a general overview and initial quantification. The second fraction consisted of fine roots included in PELCO CryO-Z-T (OCT), an embedding matrix for cryostat sectioning, in a cylindrical cast of 1.5 cm height and 1 cm diameter. The root pieces were oriented perpendicularly to the cryostat blade, to ensure transversal cuts. Serial thirty-micrometer-thick sections were obtained using a Cryostat (Leica CM 3050S). One every ten sections were placed in a microscope slide. In the end, each slide contained between 10 and 15 root sections that were stained with an acid fuchsin solution (0.01% acid fuchsin in acetic acid, ethylene glycol and lactic acid, 1:1:1, *v*/*v*/*v*) following the protocol described by Navarro-Ródenas et al. (2012a). A minimum of 100 root sections were observed per plant. According to mycorrhizal roots observed, each section was classified into one of the following mycorrhizal types: roots with extraradical hyphae, roots with intercellular hyphae, and roots with intracellular hyphae. In those cases, where no fungus was observed, the roots were classified as non-mycorrhizal. To support these observations, another portion of roots were selected and processed to make semi-thin sections of 0.5 μm, according to Gutiérrez et al. (2003), then stained with toluidine blue and observed under an Olympus BH2 microscope.

### Quantification of transcript abundance of *H. almeriense* and *T. claveryi* genes

Based on their expression levels in the transcriptomic data of *T. claveryi* and *H. almeriense* previously carried out (SRA accession No. PRJNA648328) (Marqués-Gálvez et al. 2021), some genes with critical putative functions for mycorrhizal symbiosis (see introduction section) were selected as putative molecular markers of mycorrhiza development. Similarly, reference genes for calculating relative expression of *T. claveryi* genes were designed employing transcriptomic data. We selected those genes that were highly expressed and with the smallest coefficient of variation of expression values across samples. In addition, a literature search was carried out to corroborate that the genes obtained have already been used as reference genes (housekeeping genes). Expression of root and fungal genes were determined weekly. The mycorrhizal root system was collected and washed carefully with distilled water, cut into pieces, mixed, and immediately frozen in liquid nitrogen (about 100–150 mg FW). Frozen tissues were grounded to a fine powder using a TissueLyser with glass beads (3 mm) to homogenize them. RNA was extracted with the CTAB method (Chang et al. 1993). The concentration and purity of total RNA was determined using a NanoDrop 2000c Spectrophotometer (Thermo Scientific, US). For each sample, 1 μg of total RNA was reverse transcribed using the TRANSCRIPTME RNA Kit according to manufacturer’s instructions (RT32, Blirt, Gdansk, Poland).

The sequences necessary to design plant primers were downloaded from the NCBI database (SRA accession No. PRJNA648328) or from the Mycocosm portal in the case of fungal primers. The methodology employed for designing of primers was according to Thornton and Basu (2015) using PrimerQuest software (http://www.idtdna.com/Primerquest/Home/Index), OligoAnalyzer IDT (https://www.idtdna.com/calc/analyzer) and NetPrimer (http://www.premierbiosoft.com/netprimer/) (Table 1). The transcript levels of *H. almeriense* and fungal genes were evaluated by quantitative real-time PCR (qPCR). The 10-μL reaction mixture consisted of 1.5 µl of 1:10 cDNA template, 5 µl of deionized water, 5 µl of SyBR Green Master Mix (Applied biosystems, Foster City, California, USA) and 0.6 µl of gene-specific primer mix 5 µM each. Three to five different root RNA samples for each week (biological replicates) were used for analysis, with each of them carried out in triplicate (technical replicates). Non-template controls without cDNA were used in all the PCR reactions.

**Table 1:** Candidate reference genes

Gene name	Gene ID	Primer sequence	Product size (bp)	ΔG Cross Dimer	ΔG Self- dimer	Source
*TcMAP1* (1)	1276476	5’TAGCAAAAGCGTTCAGTGGC3’ 5’GAAGGATATGCAGCGCACAC3’	70	− 2.9	0.0 − 5.2	De novo
*TcExonuclease* (1)	1142477	5’CGATGAGAGATTTGCATCCG3’ 5’GACACCTCGTCATATTCGTG3’	78	− 1.8	− 3.4 − 1.3	De novo
*TcActin (1)*	*1089750*	5’CACTGGAGCATGGGATTGT’3 5’GTACTGGATGCTCCTCAGAAAG’3				(Marqués-Gálvez et al. 2019)
*TcNiR (2)*	*1175852*	5’CTACATGGTGTCGGTTTGG3’ 5’TGTGGGCTCCTGATACTC3’	*85*	− 1	− 2.3 0.0	De novo
*TcPIN1* (2)	1084486	5’CCACAAACTCCCAAATACCG3’ 5’CCAATAATGAGAGCCAGTGC3’	146	− 1.3	0.0 0.0	De novo
*TcSSP1*(2)	1140457	5’AGCGAGGATACGAGAAATTG3’ 5’GTCCATATCACCCAGGATTTC3’	99	− 2.7	− 1.1 − 1.3	De novo
*TcEXPL* (2)	1083860	5 CCTGGGACTCAACCTATGAC3’ 5’GGGTATCCCTTGGTGTAGAG3’	91	− 3.3	0.0 0.0	De novo
*TcPME* (2)	1088896	5’CGTCAACAGCAGAGGAATG3’ 5’GTGCAAGAAGGGTATCTTGG3’	110	− 3.5	0.0 − 3.5	De novo
*TcAQP1*(2)	1292087	5’AGATCGGTTACGGCATTCAG3’ 5’CCAGGAAAATCGAAACCCTA3’				De novo
*HaTLP1* (3)	*	5’GTATTGCTGCACACAAG3’ 5’CCTGAGGGTAACTGTAAG3’	95	− 1.0	− 3.4 − 1.3	De novo
*HaPE1* (3)	*	5’ATTTCGCTCTGGATACC3’ 5’TTCCATTTCACCCTCTC3’	82	− 2.8	0.0 0.0	De novo
*HaGH1* (3)	*	5’ACCATCTAGGGTTTCAC3’ 5’CTTTGAGAACGACAACC3’	105	− 2.6	− 1.6 − 0.7	De novo
*HaAOX1* (3)	*	5’ATAGACATAGCCAAGCC3’ 5’CCTCTTCTTCCAGATACC3’	139	− 0.7	0.0 − 0.7	De novo
*HaTLP2* (3)	*	5’GAGATGGTCGGAGATATAG3’ 5’TCTTAAGGACACCTTGG3’	89	− 1.5	− 0.5 − 3.9	De novo
*HaPIP2 - 1*	*	5′ACTCCAATTGCTCTGTCC3′ 5′TTGGGTGCAATCCAGTCC3′				(Guarnizo et al. 2023)
*HaPIP2 - 7*	*	5′ATGTGTTTGTGTTGGTTTGGG3′ 5′CTCCGTTACACATTTGGGCAG3′				(Guarnizo et al. 2023)
*HaPIP2 - 11*	*	5′GAGGGAAATGAAATGCTTCTG3 5′TACTCCATCCATCCAGTCC3′				(Guarnizo et al. 2023)
*HaPIP2 - 14*	*	5′TACGGGCCGTGCTATAC3′ 5′TCCGTACATGTGGTACTTG3′				(Guarnizo et al. 2023)
*HaTIP1 - 1*	*	5′TTCTCCGGTGTTAATTAATGGTTG3′ 5′ATTGTACACAACCGGAACCAC3′				(Guarnizo et al. 2023)
*HaTIP1 - 5*	*	5′CTGAGTATGAGAGGCTTGCG3′ 5′TTCCGATCAAACTTTCCGAC3′				(Guarnizo et al. 2023)

(1) selected housekeeping genes of *T. claveryi*, (2) *T. claveryi* candidate symbiosis genes, (3) *H. almeriense* candidate symbiosis genes

For plant genes, PCR program consisted of 10 min incubation at 95 °C, followed by 40 cycles of 15 s at 95 °C and 1 min at 60 °C. Transcript levels were calculated using 2^−ΔΔCt^ method (Livak and Schmittgen 2001) to evaluate the expression of each gene. *H. almeriense* ATP synthase (AF035907.1, GenBank) was used as the plant reference gene (Marqués-Gálvez et al. 2020). For fungal genes, PCR program consisted of 10 min incubation at 95 °C, followed by 40 cycles of 15 s at 95 °C, 20 s at 62 °C and 30 s at 72 °C. Transcript levels were calculated using 2^−ΔΔCt^ method (Livak and Schmittgen 2001) to evaluate the expression of each gene normalizing gene expression to the geometric mean. The Microtubule-associated protein (ID1276476, Mycocosm) and *TcActin* (1,089,750, Mycocosm) were used as reference fungal genes, based on the results obtained in the Table S1.

### Statistical analysis

All experiments were analyzed by a one-way ANOVA, considering time as independent variable, followed by the Tukey’s HSD multiple comparison test to examine the significant differences at *P* < *0.05*. The normality and homoscedasticity of the data were checked using the Kolmogorov–Smirnov test and Levene’s test, respectively. Pearson’s correlation analysis was done using the “cor” package. All statistical analyses were performed using R and R Studio software (Martin 2021).

## Results

### Mycorrhization during EEM symbiosis formation

**Fig. 1:**
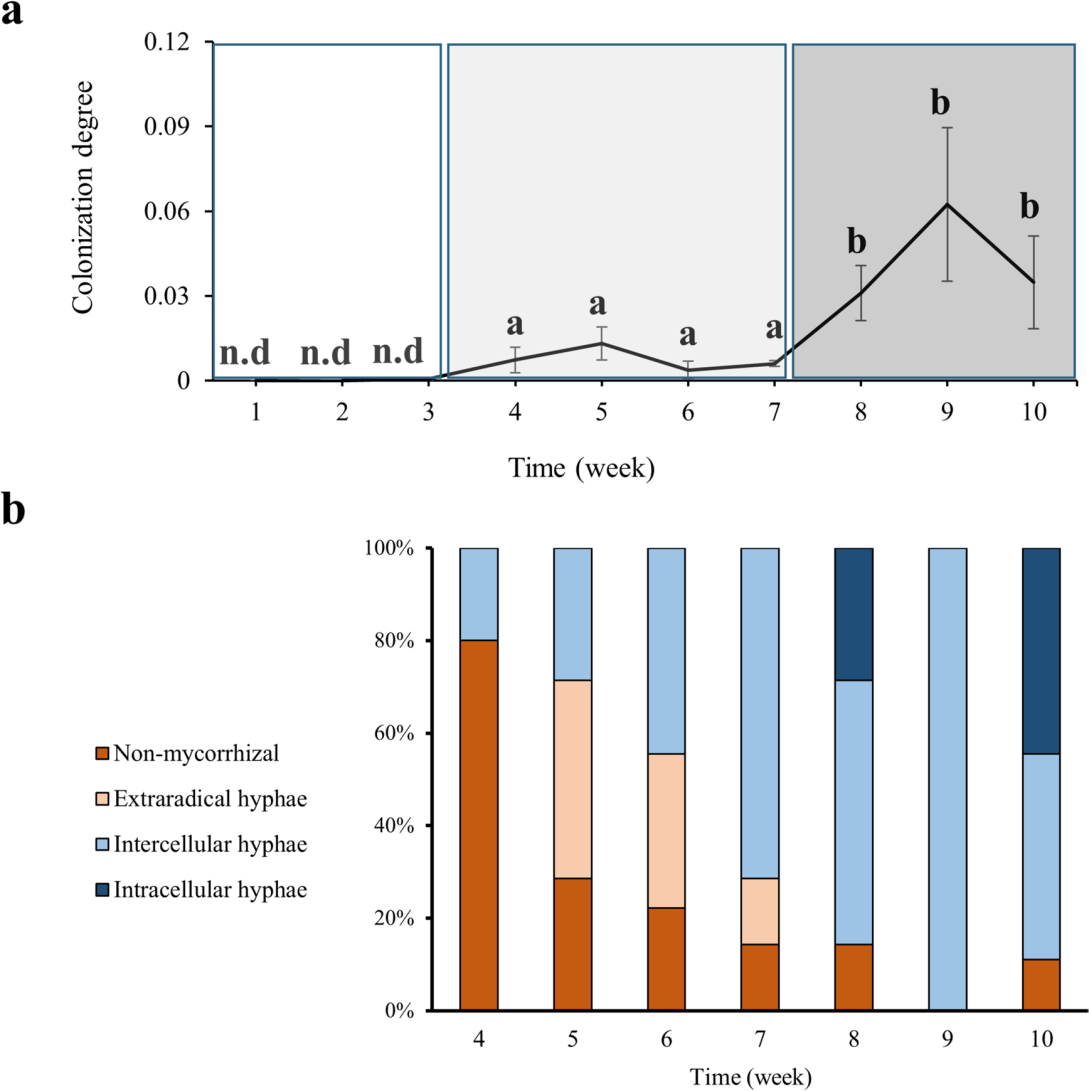
Time-course of fungal colonization of *T. claveryi* in *H. almeriense* roots. Plants were collected once per week for ten weeks. (**a**) The degree of mycorrhization was calculated as the differences in the Ct values between plant and fungi housekeeping genes (2 Ct^(HaATPsyn) − (Ct (TcActin;TcExo)^). (**b**) Characterization of fungal structures was performed via microscopy and relative abundances of each ectendomycorrhizal structure was measured during the formation of the symbiotic associations and categorized into: extraradical hyphae root, intercellular colonization root, intracellular colonization root and non-mycorrhizal root. Values represent the average ± SE (n = 5) at each sampled time point. Different letters on each time point indicate significant differences between times points (*P* < *0.05*) based on multiple comparisons (Tukey’s HSD test) in ANOVA

**Fig. 2:**
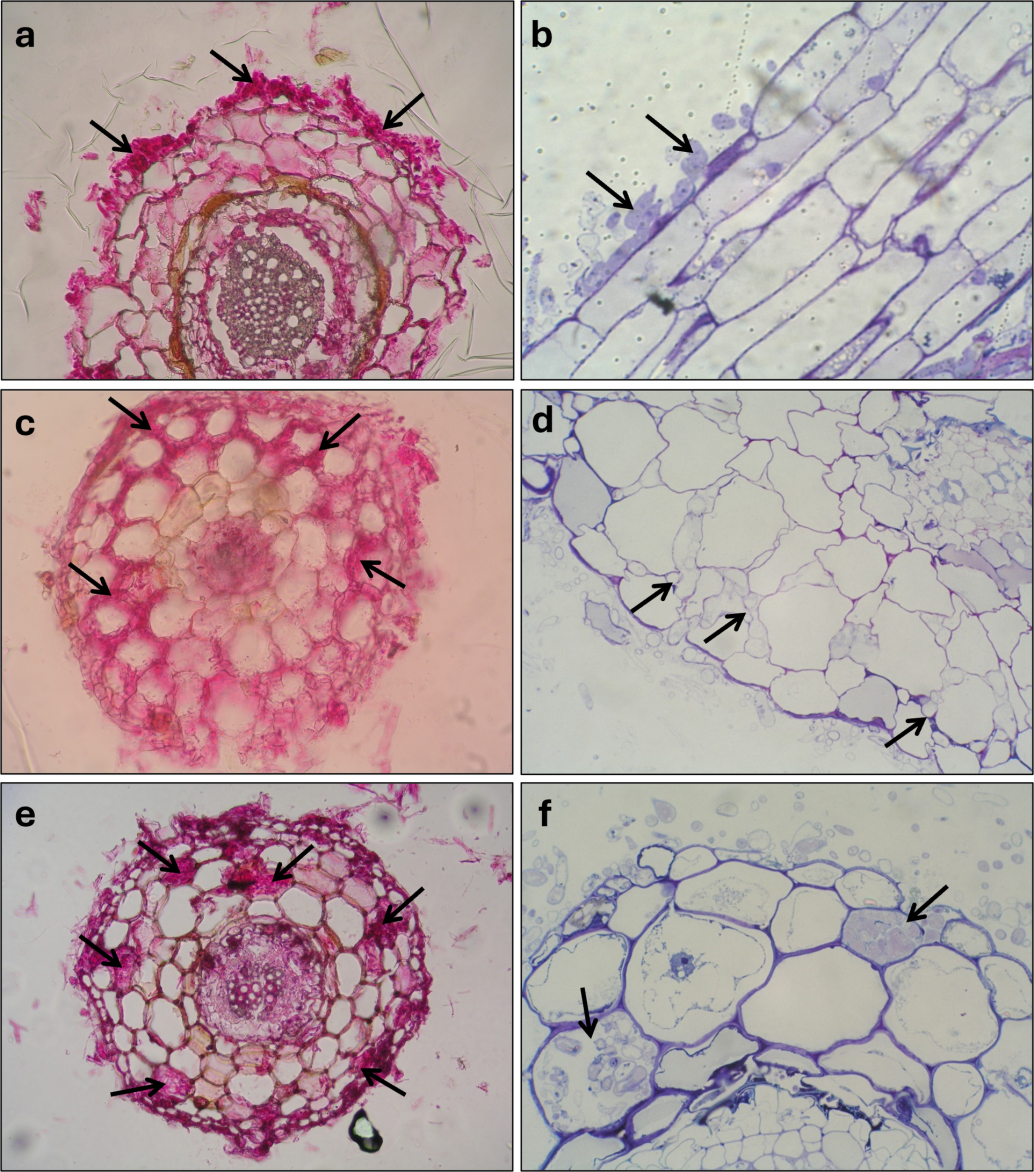
Ectendomycorrhizal *continuum* development of *T. claveryi* in *H. almeriense* roots. Black arrows indicate extraradical hyphae (**a**, **b**), intercellular hyphae forming Hartig net (**c**, **d**) and intracellular hyphae (**e**, **f**). Figs. a, c, e: cross-sections stained with acid fuchsin solution, 20X. Figs. b, longitudinal-section, d, f cross-sections: stained with toluidine blue, 40X

To follow mycorrhiza formation throughout the weeks, we coupled morphological and molecular techniques to quantify and characterize the degree of colonization during the experiment. Using microscopy and molecular tools, the presence of *T. claveryi* inoculated roots was detected from week four onwards (Fig. 1). The fungal colonizing biomass detected in *H. almeriense* roots by RT-qPCR remained constant from week four until week seven, after which it significantly increased (*P* < *0.05*) and then stayed constant until the experiment's end (Fig. 1a). In contrast, the percentage of colonized roots increased gradually up to week eight, where it stabilized and remained constant thereafter (Fig. 1b). At the morphological level, fungal hyphae structures were progressively replaced from extraradical to intercellular and from intercellular to intracellular. Over the ten weeks, a qualitative change is detected at week seven where no more extraradical mycelium is observed and the intracellular hyphae appears (Fig. 1b). According to these morphological and molecular results, three different developmental stages were identified (Fig. 2): pre-symbiosis (co-culture, presence of extraradical hyphae but not *in planta* colonization) (Figs. 2a and b), early symbiosis (progressive increase of intercellular colonization) (Figs. 2c and d) and late or mature symbiosis (stable colonization, appearance of intracellular colonization) (Figs. 2e and f). Regarding plant length and relative root surface, they increased progressively throughout the experiment, and this growth tendency reached a plateau between weeks seven and eight, fitting with the previously described mycorrhizal stages (Fig. S1).

### Selected “symbiosis toolkit” gene expression associated with stage of mycorrhizal development.

To decipher whether the expression of fungal and plant genes varies according to the established colonization stages, we followed the expression of a set of genes (Table S2) selected according to its strong regulation in *H. almeriense x T. claveryi* EEM interaction and their pertinence to mycorrhizal toolkit (Marqués-Gálvez et al. 2021) and regarding plant aquaporins regulated in Guarnizo et al. (2023).

#### Fungal gene expression

**Fig. 3:**
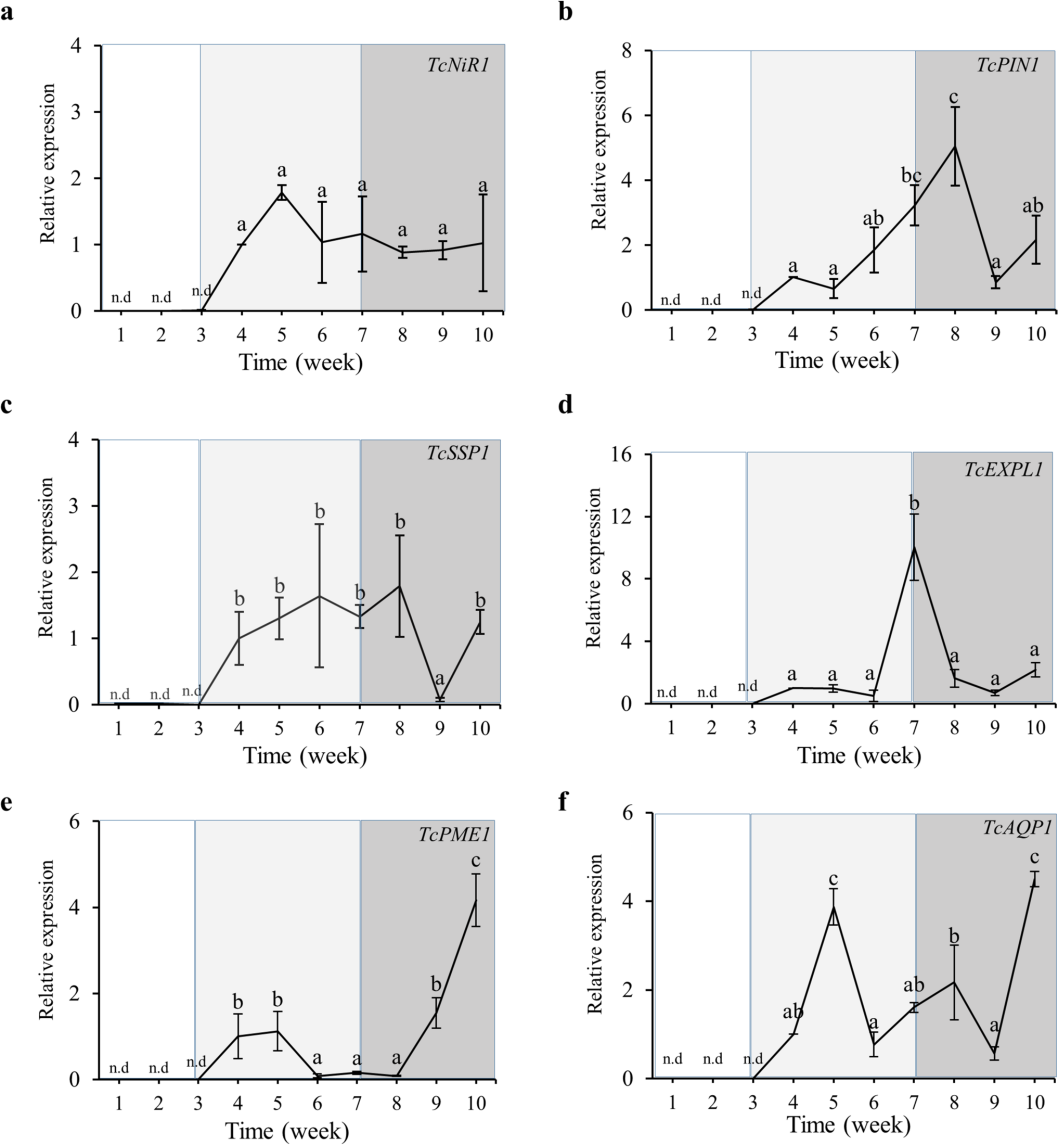
Time course expression of fungal symbiosis-induced genes in mycorrhizal *H. almeriense* plants. Plants were collected once a week, for ten weeks. For each gene, qPCR data represents fold-changes relative to the biological replicate on week 1, in which the expression was designated to be 1 and all other samples were expressed relative to it. Values represent the average ± SE (n = 5) at each sampled time point. Different letters on each time point indicate significant differences between times (*P* < *0.05*) based on multiple comparisons (Tukey’s HSD test) in ANOVA. n.d = not detected. (**a**) Nitrite reductase (*TcNiR1*), (**b**) auxin efflux carrier protein PIN-FORMED (*TcPIN1*), (**c**) small-secreted protein (*TcSSP1*), (**d**) expansin-like protein (TcEXPL1), (**e**) pectin methyl esterase (*TcPME1*), and (**f**) aquaporin (*TcAQP1*)

Upon inoculation, the transcripts of *T. claveryi* genes were monitored from the beginning of the experiment; however, transcripts were not detected inside *H. almeriense* roots until week four, corroborating our morphological analyses (Fig. 1a). Thereafter, the expressions of all *T. claveryi* candidate genes were detected and quantified, although with different expression patterns. A nitrite reductase (*TcNiR1*) showed a constant expression along the mycorrhization process (Fig. 3a). A small-secreted protein (*TcSSP*) showed a similar expression pattern, except for week 9, where a significant drop in expression was detected (Fig. 3b). Expression of an auxin efflux carrier (*TcPIN1*) and of an expansin-like protein (*TcEXPL1*) peaked at 8 and 7 weeks from the inoculation, respectively. These two peaks of expression coincide with the time of transition between early and late-symbiosis. However, this expression peak was more gradual for *TcPIN1* than for *TcEXPL1*, which had a sudden upregulation followed by a sudden drop of expression (Figs. 3c and d). Noticeably, a pectin methyl esterase (*TcPME1*) and the aquaporin (*TcAQP1*) genes followed a similar response pattern, with two well differentiated peaks in expression at early and late symbiosis (Figs. 3e and f).

#### Plant gene expression

**Fig. 4:**
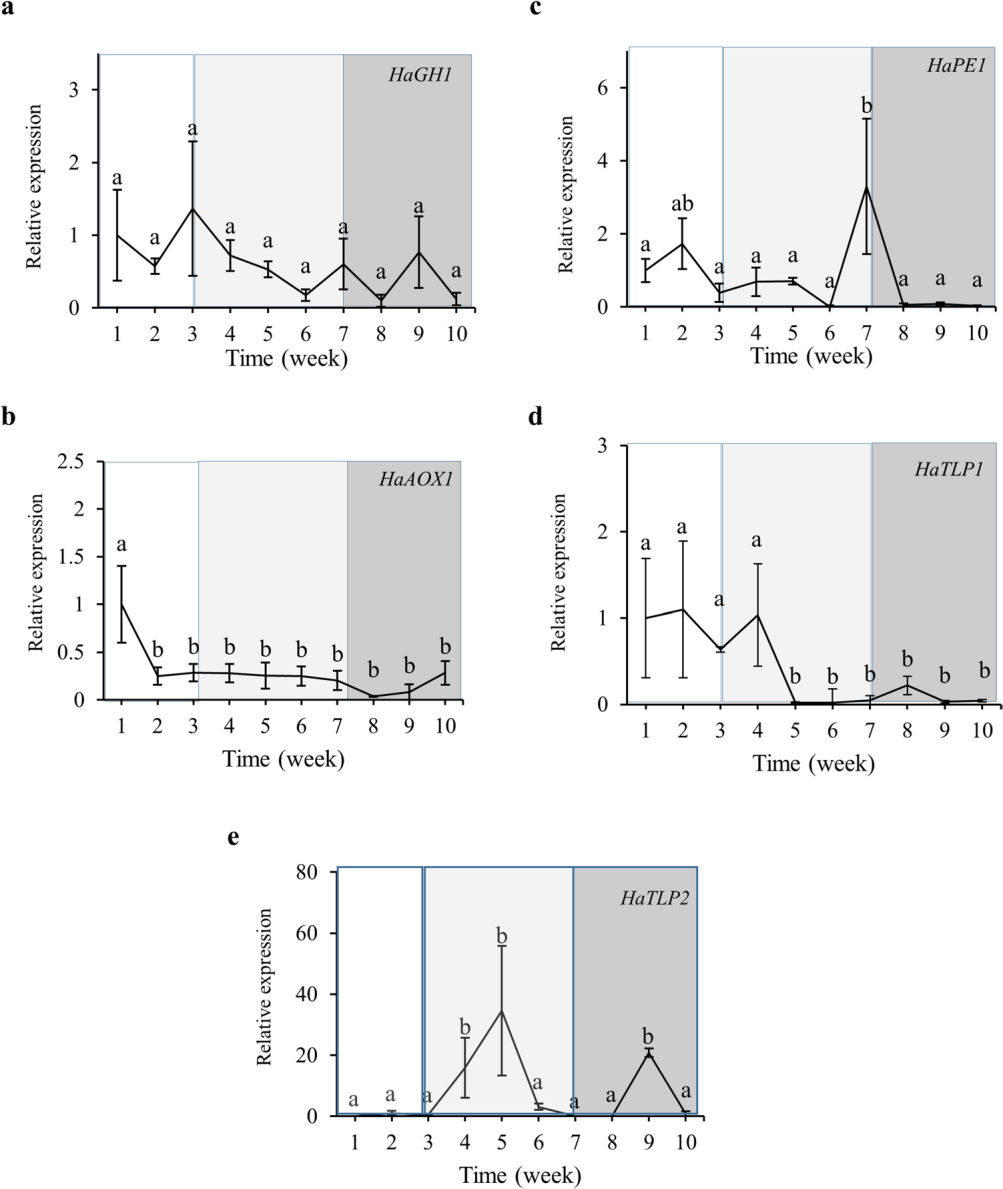
Time course expression of plants symbiosis-induced genes in mycorrhizal *H. almeriense* plants. Plants were collected once a week, for ten weeks. For each gene, qPCR data represents fold-changes relative to the biological replicate on week 1, in which the expression was designated to be 1 and all other samples were expressed relative to it. Values represent the average ± SE (n = 5) at each sampled time point. Different letters on each time point indicate significant differences between times (*P* < *0.05*) based on multiple comparisons (Tukey’s HSD test) in ANOVA. n.d = not detected. (**a**) Glycoside hydrolase (*HaGH1*), (**b**) pectin esterase (*HaPE1*), (**c**) alternative oxidase (*HaAOX1*), (**d**) thaumatin-like protein 1 (*HaTLP1*), (**e**) thaumatin-like protein 2 (*HaTLP2*)

All *H. almeriense* genes showed a significant regulation across time points, except for a glycoside hydrolase (*HaGH1*), which maintained a constant expression level (Fig. 4a). An alternative oxidase (*HaAOX1*) expression was rapidly downregulated after one week upon inoculation and remained at low levels during the whole experiment (Fig. 4b). A pectin esterase (*HaPE1*) expression reached a peak at week seven (Fig. 4c) during the transition from early to late stage. The transcripts of thaumatin-encoding genes (*HaTLPs)* showed different response patterns upon inoculation. *HaTLP1* showed a remarkable downregulation response in the transition from pre-symbiosis to early stage (about twofold decrease) (Fig. 4d), whereas the transcriptional response of the *HaTLP2* reached two upregulation peaks at five and nine weeks (Fig. 4e).

### Correlation analysis

Using only data from week four onwards, we combined all gene expression data and then correlated the gene expression with the degree of colonization. We analyzed separately between early stage and late stage. During the early stage, two negative significant correlations between *TcEXPL1* or *TcPIN1* and the degree of colonization was found (Table 2). However, during the late stage, no genes expression were significantly correlated with colonization.

**Table 2:** Pearson’s correlation of early stage and late-stage gene expressions with degree of colonization

Type		*TcNiR1*	*TcPIN1*	*TcSSP1*	*TcEXPL1*	*TcPME1*	*TcAQP1*
Early stage	R2 p-value	− 0.18 0.59	− 0.61 0.04	− 0.16 0.62	− 0.68 0.019	− 0.06 0.86	− 0.18 0.57
Late stage	R2 p-value	0.43 0.30	0.00 1.00	− 0.02 0.97	− 0.52 0.16	− 0.03 0.95	− 0.27 0.49
Type		HaTLP1	HaPE1	HaAOX1	HaGH1	HaTLP2	
Early stage	R2 p-value	− 0.47 0.15	− 0.042 0.9	− 0.16 0.65	− 0.39 0.24	− 0.23 0.50	
Late stage	R2 p-value	− 0.07 0.88	0.067 0.88	− 0.5 0.18	0.66 0.053	− 0.10 0.81	

### Plant aquaporin expression during mycorrhizal development

**Fig. 5:**
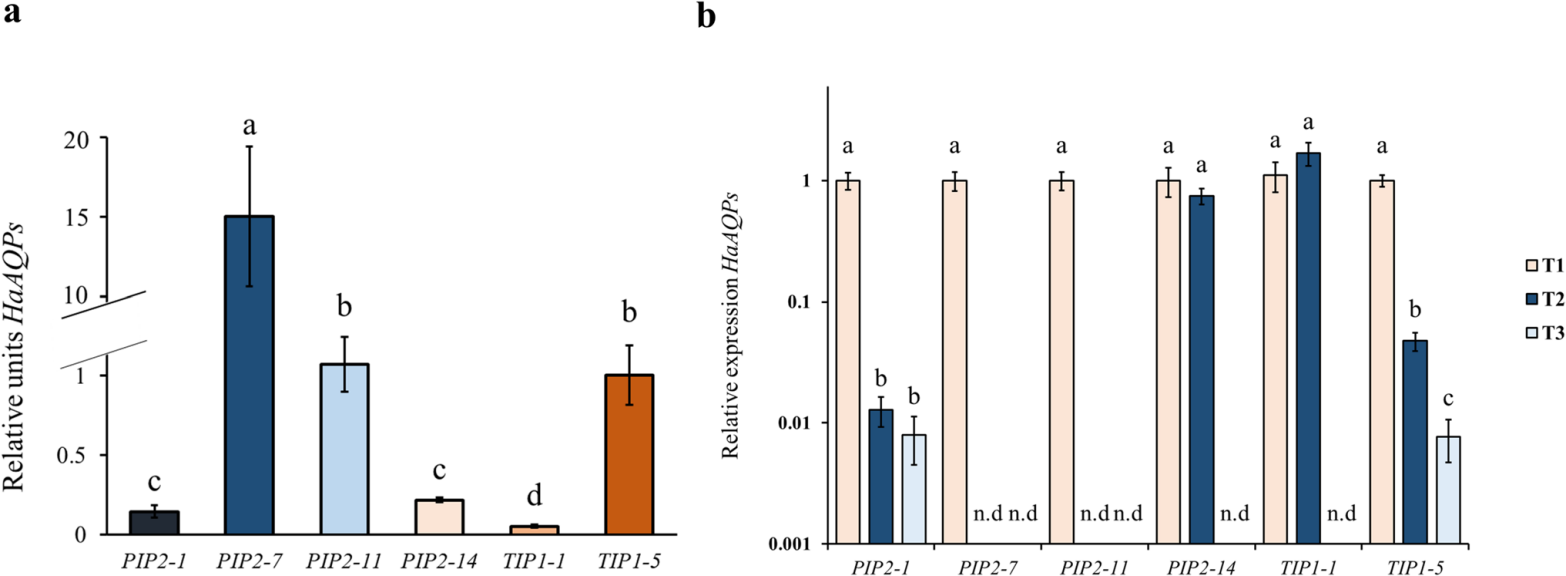
Time course expression of plant aquaporins in mycorrhizal *H. almeriense* plants. (**a**) Relative expression levels (relative units) of the *HaAQPs* in roots on pre-symbiosis stage (week one). (**b**) Individual HaAQPs expressions in roots during three different points: Pre-symbiosis (week one), early symbiosis (week five) and late symbiosis (week ten). For each aquaporin, qPCR data represents fold-changes relative to the biological replicate on pre-symbiosis, in which the expression was designated to be 1 and all other samples were expressed relative to it. Bars represent the average ± SE (n = 5). Different letters on the bars indicate significant differences (*P* < *0.05*) based on multiple comparisons (Tukey’s HSD test) in ANOVA. n.d. = not detected

Apart from the analysis of the RNAseq-selected plant and fungal genes (Marqués-Gálvez et al. 2021), we also used RT-qPCR to evaluate relative plant AQP expression at three time points coinciding with the different mycorrhizal stages: week one (pre-symbiosis), five (early stage) and ten (late stage) weeks upon inoculation (Fig. 5). Firstly, we measured the expression of *HaAQPs* at pre-symbiosis and observed differences (*P* < *0.05*) in *HaAQPs* expression among the six selected genes (Fig. 5a). The *HaTIP1-1* expression was the least expressed, while *HaPIP2-7* expression was the most highly expressed. Furthermore, we confirmed that each AQP had a different expression profile during mycorrhizal development (Fig. 5b). *HaAQPs* expression was significantly downregulated at late stage (*P* < *0.05*) when compared to pre-symbiosis in all the cases. At early stage, they were all downregulated except for *HaPIP2-14* and *HaTIP1-1,* which expressions did not change. Of note, these AQPs were already poorly expressed compared to the rest of AQPs at pre-symbiotic stage.

## Discussion

Over the experimental procedure period, the development of the ectendomycorrhizal symbiosis between *H. almeriense* and *T. claveryi* can be divided into three stages in base of the structure and colonization degree: the pre-symbiotic stage, early symbiosis stage and late symbiosis stage, each of them with their own traits.

### The pre-symbiotic stage of *H. almeriense* x *T. claveryi* lasts three weeks in pot conditions

We did not detect presence of the fungus via microscopy, nor by RT-qPCR in the plant until the fourth week, suggesting that the pre-symbiotic stage of *H. almeriense* x *T. claveryi* EEM lasted around three weeks. In this period, it is plausible that *T. claveryi* was establishing in the rhizospheric soil interacting with the roots of *H. almeriense*. The lack of data from *T. claveryi* gene expression in soil prevents us to evaluate the expression of any of the selected fungal genes as molecular markers of this stage. However, mycorrhizal development is a dynamic process, and not all the hyphae enters direct plant-fungal contact with the roots simultaneously. In weeks four to eight, between 10 and 40% of hyphae observed were still extraradical and, thus, this data could also be used as a proxy of the genetic regulation before plant-fungal direct contact. According to this, the progressive increase of *TcNiR*, *TcSSP* and *TcPIN1* levels from week four to eight may suggest a role also in the pre-symbiotic stage. Among these three genes, the putative role of *TcPIN1* remains of particular interest, since auxin of fungal origin (indole- 3-acetic acid or IAA) is one of the most important phytohormones that remodels root architecture and facilitate fungal accommodation (Felten et al. 2009; Sukumar et al. 2013; Fusconi 2014). Our results regarding *TcPIN1* levels are in accordance with previous reports showing that *Terfezia* spp. produce IAA, and this plays a role in the dynamics of the EEM *continuum* (Zaretsky et al. 2006a; Sitrit et al. 2014; Turgeman et al. 2016). From the plant side, most of the gene expressions remain stable during pre-symbiotic stage, except for *AOX1*. It has been reported that non-mycorrhizal plants increase the production of *AOX* under stressful conditions to eliminate reactive oxygen species (ROS) (Vanlerberghe 2013; Li et al. 2013). Although the role of AOX has not been fully elucidated yet, evidence suggests that AOX function in metabolic and signaling homeostasis is particularly important during stress. The roots of *H. almeriense* exhibit high levels of *AOX1* just during the first week only, but its expression rapidly declines. This decrease in *AOX1* expression could be comparable to the AM-suppression of AOX activity previously reported (Liu et al. 2015; Del-Saz et al. 2018), although its biological insights remain unresolved. These results could also be coupled with the progressive resolution of stress conditions originated by transplant or substrate manipulation during fungal inoculation. In either case, more dedicated experiments with proper controls would need to be performed to unveil the putative role of *H. almeriense* AOX during pre-symbiotic stage.

### The early symbiotic stage is characterized by intercellular colonization and the upregulation of several marker fungal genes of colonization

At this stage, mycorrhization can be detected both at the molecular and microscopic levels and takes four weeks. During this period the percentage of colonized roots increases linearly from 20 to 70%. However, the ratio of fungal biomass over roots biomass remains constant. During the early symbiotic phase, all root colonization occurs intercellularly, and the fungus appears to prioritize spreading along the whole system of fine roots rather than establishing itself. Even as the percentage of colonized roots increases, fungal biomass tends to become diluted within them as mycorrhization development progresses, we find more roots with intercellular colonization and less extraradical mycelium. Regarding the expression of molecular markers *TcPIN1*, *TcNiR*, *TcSSP* and *TcAQP1* seem to play an important role at this stage, according to their expression profiles.

The possible implications of *TcPIN1* have been discussed in the previous phase, while those of TcAQP1 are discussed in Sect."The role of AQPs in mycorrhizal symbiosis". *TcNiR* shows the most stable upregulated pattern from all the selected genes, making it probably the best candidate to be used as mycorrhization marker, regardless of its developmental stage. TcSSP expression levels are also high during this phase. Fungal effectors are normally orphan genes, with low-sequence homology (Kohler et al. 2015), preventing us from inferring its function by homology. However, its expression pattern is similar to those of previously characterized fungal effectors in other ECM species, that facilitate the fungal colonization by interfering with the plant immune system, such as *LbMiSSP7* (Plett et al. 2014). The expression pattern of defense-related *HaTLP1* may support this same role for *TcSSP*, since it shows a marked decline at early symbiosis stage, coinciding with the start of colonization and the upregulation of *TcSSP.* However, this was not the case for another defense-related gene *HaTLP2*. Recent research has shown that *LbMiSSP7* is capable of interfering with the activity of poplar (*Populus tremula* × *alba)* defense master regulator *MYC2* and thus maintain the repression over specific pathways of plant jasmonate-related defense, but not for others (Marqués-Gálvez et al. 2024). This could explain the different pattern of expression between *HaTLP1* and *HaTPL2,* but more in-depth studies are needed to evaluate this.

### Intracellular colonization is the hallmark of the late symbiotic stage and it is accompanied by the expression of fungal and plants cell-wall remodelling genes

At this stage, there is an increase in the percentage of mycorrhization (reaching its maximum) and changes in fungal biomass and in mycorrhiza morphology, since fine roots with intracellular colonization were firstly observed and increased progressively in percentage. The concomitant increase in intracellular colonization and fungal biomass suggests that intracellular colonization is denser, containing more biomass per gram of root than intercellular colonization. Thus, mycorrhization moves from a phase of expansion of new roots to a phase of settlement and maturation within the root system. Due to the method used to analyze the morphology of the roots, we could not determine if the transition from inter to intracellular colonization follows a direction from tip to base, as suggested for *Pinus* spp. x *W. mikolae* EEM (Yu et al. 2001). Although our results suggest that this transition is homogenous within the whole root system, in the future, it would be interesting to determine whether the presence of intracellular colonization follows a local gradient or if it is correlated to the maturity degree of the host root.

The presence of the first intracellular hyphae coincided with an interesting pattern of expression of both fungal and plant cell wall remodeling genes *TcPME1*, *TcEXPL1* and *HaPE1.* Their expression profiles suggest their involvement in the transition from early to mature mycorrhiza, although in different ways. Previous reports have proposed a role for pectin methyl esterases in the growth of hyphae within the roots (Chowdhury et al. 2022), which is in line with the expression pattern observed for *TcPME1*. The progressive upregulation of *TcPME1* could be related both to the increase of the degree of colonization and to the apparition of intracellular colonization. Both *TcEXPL1* and *HaPE1* present a prominent peak of expression at week 7, during the transition phase from fully ecto to ectendomycorrhiza. Expansins are proteins involved in cell wall loosening and increasing cell wall extensibility (Kerff et al. 2008; Georgelis et al. 2014), and for example, in tomato roots, they are considered a prerequisite for the accommodation of the fungus in the plant (Dermatsev et al. 2010). Pectin esterases play a fundamental role in remodeling plant cell walls and have also been shown to play a role in plant defense (Micheli 2001). As intracellular colonization appears, both genes could serve as markers of the ecto to endo transition, either because of their role in plant cell wall remodeling, or as a defense response to the more intimate intracellular colonization. The further characterization of these genes could shed light to the molecular mechanisms related to the plant cell wall remodeling activities needed to accommodate fungi intracellularly.

During the symbiosis between *T. boudieri* and *C. incanus* roots, an endogenous application of a synthetic auxin produced a change from intercellular to intracellular colonization (Zaretsky et al. 2006b). In our study, the auxin efflux carrier *TcPIN1*, which reached its maximum peak during intracellular colonization, supporting the role of auxin as a determinant of the ecto to endo switch of *Terfezia* spp. EEM. While its expression drops sharply, high auxin levels have been shown to lead to reduced root cell elongation, and this attenuation in taproot growth may contribute to the equalization of growth rates between the fungus and the host root, thereby enhancing the likelihood of successful mycelial-root interaction (Campanoni and Nick 2005; Turgeman et al. 2016).

### The role of AQPs in mycorrhizal symbiosis

We also observed important transcription profiles for both fungal and plant aquaporins. *TcAQP1* is the sole gene encoding for AQP in *T. claveryi* genome (Marqués-Gálvez et al. 2021) and, thus, we could hypothesize that this gene plays multipurpose roles depending on the mycorrhization stage in which *T. claveryi* is found. In fact, the double expression peak observed for *TcAQP1* in early and late symbiosis stage supports this contention. This AQP is known to facilitate de passage of both water and CO_2_ (Navarro-Ródenas et al. 2012b) which can act as a signaling molecule in various fungal processes, including growth, differentiation, ascocarps development and mycorrhiza development (Bahn and Mühlschlegel 2006; Navarro-Ródenas et al. 2015; Xu et al. 2016). In *L. bicolor*, the upregulation of AQPS is pivotal for the transition from pre-symbiotic phase to functional symbiosis (Navarro-Ródenas et al. 2015). Whereas in the early phase it could be favoring the transport of signaling molecules, as it has been suggested for its homologue *LbAQP1* (Navarro-Ródenas et al. 2015), at the end of this stage, when the mycorrhiza is already mature and the molecular signaling is less intense, functions related to water transport are expected (Navarro-Ródenas et al. 2012b). Regarding the expression profile of plant AQPs, we found a general downregulation of 6 different genes throughout the course of the mycorrhization development. This can be related to molecular reprogramming in response to the presence of the fungus. We can hypothesize that one of the reasons of this phenomenon is that younger and non-mycorrhizal plants are more dependent on their own water transport systems, while more adult mycorrhizal plants may rely more on the water-acquisition benefits provided by *T. claveryi* colonization (Navarro-Ródenas et al. 2013). More dedicated analyses evaluating the whole transcriptional landscape of plant AQPs will be needed to determine which *H. almeriense* AQP isoforms play a role during mycorrhizal development.

### Conclusions

In this work we report the ectendomycorrhizal *continuum* development in *T. claveryi* x *H. almeriense* in pot conditions, revealing three well defined stages. Each stage is characterized by different morphological structures, with no fungal colonization in presymbiosis stage, exclusively intercellular colonization in early stages of the interaction and concomitant inter- and intracellular colonization during the late stage of symbiosis. The expression of certain molecular markers during these stages helps us better understand the mechanisms of EEM symbiosis development. In the future, more refined molecular tools for the *H. almeriense* x *T. claveryi* system together with more in-depth analyses are needed to clarify the function of the pinpointed marker genes in the establishment of EEM symbiosis.

## Supplementary Information

Below is the link to the electronic supplementary material.

Supplementary file1 (DOCX 168 KB)

## Data Availability

No datasets were generated or analysed during the current study.
