# Supplementary material for: Morphological and molecular development of Terfezia claveryi ectendomycorrhizae exhibits three well-defined stages
Source: Mycorrhiza. 2025 Apr 15;35(2):31. doi: 10.1007/s00572-025-01205-8 (PMC12000269; doi:10.1007/s00572-025-01205-8)
Supplement: Supplementary file 1 — Supplementary file1 (DOCX 168 KB) [file 572_2025_1205_MOESM1_ESM.docx]

**Supplementary Data**

To test specificity and cross-amplification, all selected primers were tested on two physiological stages of *T. claveryi*, ascocarp and mycelium. In addition, real-time PCR experiments were carried out in six separate biological samples and non-template controls were performed in all PCR reactions. The expression stability of selected housekeeping genes was calculated using various bioinformatics tools: Delta Ct (Livak and Schmittgen 2001), BestKeepeer (Pfaffl et al. 2004), NormFinder (Andersen et al. 2004), geNorm (Vandesompele et al. 2002), these tools will allow us to obtain a ranking of which genes are more stable, and each one will use its algorithm for this. Finally, we will use RefFinder (Xie et al. 2012), an algorithm that integrates the results obtained from the previous bioinformatics tools and then calculating a final ranking based on the geometric mean for each gene (**Table S1)**

**Table S1.** Expression stability ranking of the ten candidate reference genes according to geNorm, BestKeeper, NormFinder, Delta CT, and the RefFinder comprehensive analysis. These analyses were performed using the web-based RefFinder tool found at [www.heartcure.com.au/reffinder/](http://www.heartcure.com.au/reffinder/) .

| Gene | **RefFinder** | | **Delta Ct** | | **geNorm** | | **NormFinder** | | **Bestkeeper** | |
| --- | --- | --- | --- | --- | --- | --- | --- | --- | --- | --- |
|  | Geomean of ranking values | Ranking | ST  dev | Ranking | M  value | Ranking | Stability  value | Ranking | SD | Ranking |
| *TcRho* | 2.00 | 1 | 2.00 | 2 | 0.34 | 1 | 0.68 | 2 | 0.95 | 4 |
| *TcMAP1* | 2.06 | 2 | 2.04 | 3 | 0.34 | 1 | 0.86 | 3 | 0.71 | 2 |
| *TcNucleosome* | 2.24 | 3 | 1.99 | 1 | 1.24 | 5 | 0.40 | 1 | 1.41 | 5 |
| *TcActin* | 2.78 | 4 | 2.26 | 4 | 0.67 | 2 | 1.47 | 6 | 0.54 | 1 |
| *TcExonuclease* | 4.74 | 5 | 2.64 | 6 | 0.86 | 3 | 1.90 | 7 | 0.82 | 3 |
| *TcTropomyosine* | 5.18 | 6 | 2.55 | 5 | 1.77 | 5 | 0.92 | 4 | 2.18 | 6 |
| *TcATPase* | 6.74 | 7 | 2.80 | 7 | 2.11 | 6 | 1.39 | 5 | 2.59 | 7 |
| *TcMAFP1* | 8.00 | 8 | 3.75 | 8 | 2.51 | 7 | 2.61 | 8 | 3.47 | 8 |

**Table S2**. List of genes selected from both plants and fungi for gene expression assay.

| **Gene description** | **Gene name** | **Gene ID** | **Selection criteria** |
| --- | --- | --- | --- |
| Nitrite reductase | *TcNiR* | *1175852* | Top 1 upregulated *T. claveryi* gene in mycorrhizal roots vs free living mycelium (Marqués-Gálvez et al., 2021). It may play a pivotal role in nitrogen assimilation and cycling (Pena. 2017). |
| Auxin efflux carrier protein PIN-FORMED | *TcPIN1* | 1084486 | Upregulated *T. claveryi* gene in response to mycorrhizal symbiosis and mycorrhizal response to drought stress, where a shift from ecto to ectendomycorrhizal structures occurs (Marqués-Gálvez et al., 2021). Auxin signaling plays a crucial role in the modification of root growth during ectomycorrhizal symbiosis formation (Felten et al. 2009) and influences ectendomycorrhizal relationships (Zaretsky et al.2006) |
| Small-secreted protein | *TcSSP1* | 1140457 | Top 20 upregulated *T. claveryi* gene in mycorrhizal roots vs free living mycelium (Marqués-Gálvez et al. 2021). It’s the most upregulated putative fungal effector, which comprises a group of orphan small secreted proteins that may play critical roles during mycorrhiza establishment (Kohler et al. 2015). |
| Expansin-like protein | *TcEXPL* | 1083860 | Upregulated *T. claveryi* gene in mycorrhizal roots vs free living mycelium (Marqués-Gálvez et al. 2021). Proteins involved in cell wall loosening may be considered a prerequisite for the accommodation of the fungus in the plant (Veneault-Fourrey et al. 2014). |
| Pectin methyl esterase | *TcPME* | 1088896 | Upregulated *T. claveryi* gene in mycorrhizal roots vs free living mycelium and downregulated in mycorrhizal response to drought stress, where a shift from ecto to ectendomycorrhizal structures occurs (Marqués-Gálvez et al. 2021). Studies in *Laccaria bicolor* have recently highlighted the importance of pectin methylesterases in Hartig net formation (Chowdhury et al. 2022). |
| Aquaporin | *TcAQP1* | 1292087 | Top 100 upregulated *T. claveryi* gene in mycorrhizal roots vs free living mycelium (Marqués-Gálvez et al. 2021). Previously characterized as water, small solutes and CO_2_ transporter (Navarro-Ródenas et al. 2013). *L. bicolor* AQP1 may play a critical role during ectomycorrhizal establishment (Navarro-Ródenas et al. 2015). |
| Thaumatin-like protein | *HaTLP1* | * | Top 20 upregulated *Helianthemum almeriense* gene in mycorrhizal vs non mycorrhizal roots (Marqués-Gálvez et al. 2021). Thaumatin-like proteins are a group of proteins that are induced in plants in response to infection by pathogens, elicitors and/or stress factors (Montero et al. 2003). |
| Pectin esterase | *HaPE1* | * | *H. almeriense* downregulated gene in mycorrhizal vs non-mycorrhizal roots, both in well-watered and drought conditions (Marqués-Gálvez et al. 2021). Plant CAZymes may play a role in fungal accommodation (Gong et al. 2023). |
| Glycoside hydrolase | *HaGH1* | * | *H. almeriense* downregulated gene in mycorrhizal vs non-mycorrhizal roots, both in well-watered and drought conditions (Marqués-Gálvez et al. 2021). Plant CAZymes may play a role in fungal accommodation (Gong et al. 2023). |
| Alternative oxidase | *HaAOX1* | * | *H. almeriense* downregulated gene in Mycorrhizal vs non-mycorrhizal roots, both in well-watered and drought conditions (Marqués-Gálvez et al., 2021). Previous studies of arbuscular mycorrhiza colonization show suppression on AOX activity (Liu et al. 2015) |
| Thaumatin-like protein | *HaTLP2* | * | Top 20 upregulated Helianthemum almeriense gene in mycorrhizal vs non mycorrhizal roots (Marqués-Gálvez et al. 2021). Thaumatin-like proteins are a group of proteins that are induced in plants in response to infection by pathogens, elicitors and/or stress factors (Montero et al. 2003). |


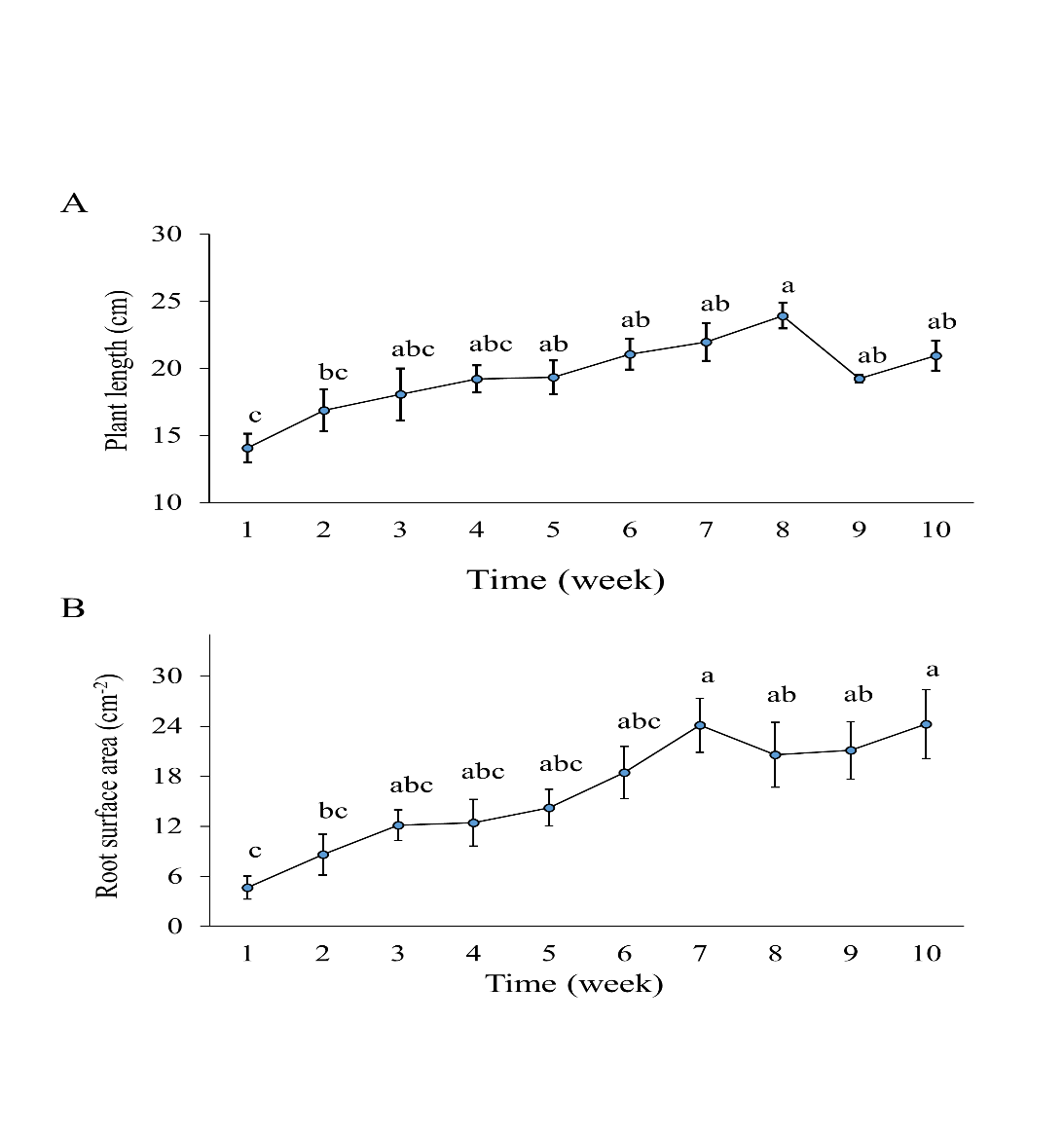


**Figure S1. Time course development of plants symbiosis-induced genes in mycorrhizal *H. almeriense plants*.** Plants were collected once for ten weeks. A) Total plant length. B) Relative root surface. Values represent the means ± SE (n=5) at each sampled time point. Different letters on each time point indicate significant differences between times (*P < 0.05*) based on multiple comparisons (Tukey’s HSD test) in ANOVA.

**References**

Andersen CL, Jensen JL, Ørntoft TF (2004) Normalization of real-time quantitative reverse transcription-PCR data: A model-based variance estimation approach to identify genes suited for normalization, applied to bladder and colon cancer data sets. Cancer Research 64:5245–5250. https://doi.org/10.1158/0008-5472.CAN-04-0496

Chowdhury J, Kemppainen M, Delhomme N, et al (2022) *Laccaria bicolor* pectin methylesterases are involved in ectomycorrhiza development with *Populus tremula* x *Populus tremuloides*. New Phytologist 236:639–655

Felten J, Kohler A, Morin E, et al (2009) The Ectomycorrhizal Fungus *Laccaria bicolor* Stimulates Lateral Root Formation in *Poplar* and *Arabidopsis* through Auxin Transport and Signaling. Plant Physiology 151:1991–2005. https://doi.org/10.1104/pp.109.147231

Gong Y, Lebreton A, Zhang F, Martin F (2023) Role of carbohydrate-active enzymes in mycorrhizal symbioses. Essays in Biochemistry 67:471–478

Kohler A, Kuo A, Nagy LG, et al (2015) Convergent losses of decay mechanisms and rapid turnover of symbiosis genes in mycorrhizal mutualists. Nature Genetics 47:410–415. https://doi.org/10.1038/ng.3223

Liu Z, Li Y, Wang J, et al (2015) Different respiration metabolism between mycorrhizal and non-mycorrhizal rice under low-temperature stress: A cry for help from the host. Journal of Agricultural Science 153:602–614. <https://doi.org/10.1017/S0021859614000434>

Livak KJ, Schmittgen TD (2001) Analysis of relative gene expression data using real-time quantitative PCR and the 2-ΔΔCT method. Methods 25:402–408. https://doi.org/10.1006/meth.2001.1262

Marqués-Gálvez JE, Miyauchi S, Paolocci F, et al (2021) Desert truffle genomes reveal their reproductive modes and new insights into plant–fungal interaction and ectendomycorrhizal lifestyle. New Phytologist 229:2917–2932. https://doi.org/10.1111/nph.17044

Monteiro, S, Barakat, M, Piçarra-Pereira, MA, et al (2003). Osmotin and thaumatin from grape: a putative general defense mechanism against pathogenic fungi. Phytopathology, 93:1505-1512.

Pena, R. (2016). Nitrogen acquisition in ectomycorrhizal symbiosis. Molecular mycorrhizal symbiosis, 179-196. <https://doi.org/1002/9781118951446.ch11>

Pfaffl MW, Tichopad A, Prgomet C, Neuvians TP (2004) Determination of stable housekeeping genes, differentially regulated target genes and sample integrity: BestKeeper - Excel-based tool using pair-wise correlations. Biotechnology Letters 26:509–515. https://doi.org/10.1023/B:BILE.0000019559.84305.47

Vandesompele J, De Preter K, Pattyn F, et al (2002) Accurate normalization of real-time quantitative RT-PCR data by geometric averaging of multiple internal control genes. Genome biology 3:. https://doi.org/10.1186/gb-2002-3-7-research0034

Veneault-Fourrey C, Commun C, Kohler A, et al (2014) Genomic and transcriptomic analysis of *Laccaria bicolor* CAZome reveals insights into polysaccharides remodelling during symbiosis establishment. Fungal Genetics and Biology 72:168–181. <https://doi.org/10.1016/j.fgb.2014.08.007>

Xie F, Xiao P, Chen D, et al (2012) miRDeepFinder: A miRNA analysis tool for deep sequencing of plant small RNAs. Plant Molecular Biology 80:75–84. https://doi.org/10.1007/s11103-012-9885-2

Zaretsky M, Sitrit Y, Mills D, et al (2006) Differential expression of fungal genes at preinfection and mycorrhiza establishment between *Terfezia boudieri* isolates and Cistus incanus hairy root clones. New Phytologist 171:837–846. https://doi.org/10.1111/j.1469-8137.2006.01791.x
